# Long COVID-19 Pathophysiology: What Do We Know So Far?

**DOI:** 10.3390/microorganisms11102458

**Published:** 2023-09-30

**Authors:** Nikolaos-Renatos Tziolos, Petros Ioannou, Stella Baliou, Diamantis P. Kofteridis

**Affiliations:** 1 Department of Internal Medicine & Infectious Diseases, University Hospital of Heraklion, 71110 Heraklion, Greecekofterid@uoc.gr (D.P.K.); 2 School of Medicine, University of Crete, 71003 Heraklion, Greece

**Keywords:** COVID-19, post-COVID-19, Long COVID-19, SARS-CoV-2

## Abstract

Long COVID-19 is a recognized entity that affects millions of people worldwide. Its broad clinical symptoms include thrombotic events, brain fog, myocarditis, shortness of breath, fatigue, muscle pains, and others. Due to the binding of the virus with ACE-2 receptors, expressed in many organs, it can potentially affect any system; however, it most often affects the cardiovascular, central nervous, respiratory, and immune systems. Age, high body mass index, female sex, previous hospitalization, and smoking are some of its risk factors. Despite great efforts to define its pathophysiology, gaps remain to be explained. The main mechanisms described in the literature involve viral persistence, hypercoagulopathy, immune dysregulation, autoimmunity, hyperinflammation, or a combination of these. The exact mechanisms may differ from system to system, but some share the same pathways. This review aims to describe the most prevalent pathophysiological pathways explaining this syndrome.

## 1. Introduction

Four years after the entry of severe acute respiratory syndrome coronavirus 2 (SARS-CoV-2) into our lives, knowledge of its pathophysiology is still increasing daily. Long Coronavirus Disease 2019 (COVID-19) is a well-recognized clinical entity and significant public health issue affecting millions of people worldwide, limiting their daily activities [1]. Although it has received various names in the literature (post-COVID-19 syndrome, long-haul COVID-19, post-acute COVID-19, long-term effects of COVID-19, chronic COVID-19, post-acute sequelae of SARS-CoV-2), it remains one clinical entity, defined as the appearance of new related symptoms and signs after initial infection with SARS-CoV-2 or the persistence of symptoms after SARS-CoV-2 infection. These symptoms may persist for over four weeks, relapse, or progress [2]. In particular, signs of post-COVID-19 syndrome last from three up to twelve weeks, while clinical manifestations of chronic COVID-19 are observed beyond twelve weeks [3,4].

Long COVID-19 is associated with at least 65 million cases [5], accompanied by an estimated cost of USD 4 trillion in the US [6]. Notably, SARS-CoV-2 may affect up to 35% of outpatients and up to 87% of those hospitalized, and is more predominant in those with advanced older age and comorbidities [7]. Such comorbidities are metabolic or endocrine disturbance, type 2 diabetes mellitus (T2DM), cardiovascular symptoms, hypertension and dyslipidemia [8].

The most commonly reported symptoms are cognitive dysfunction, known as “brain fog”, joint, chest, and muscle pains, shortness of breath, anosmia, hair loss, sneezing, reduced libido and, finally, fatigue (53.1%), which is the most prevalent [9]. Unexpectedly, the majority of individuals with long COVID-19 deal with post-exertional symptom exacerbation (PESE) [10], as well as myalgic encephalomyelitis/chronic fatigue syndrome (ME/CFS) [10].

Various risk factors have been found to strongly predict the onset of post-COVID-19 syndrome. For example, female sex, smoking, comorbidities, and obesity are some risk factors [11]. However, it can affect almost every organ of the human body, predominantly the cardiovascular and nervous systems, and may also cause thrombotic events [5]. In a recent meta-analysis, female sex, age, smoking, high body mass index (BMI), age above thirty, comorbidities, and previous hospitalization or intensive care unit (ICU) admission were found to increase the risk of developing long COVID-19. On the contrary, two doses of the COVID-19 vaccine were found to hinder the long-term effects of COVID-19 [12].

The exact pathophysiological mechanisms of this syndrome are yet to be defined. The most common underlying mechanisms are immune dysregulation that leads to viral persistence, microbiota disruption, autoimmunity, endothelitis, metabolic dysregulation, and post-intensive care syndrome [5,7,13]. Data from the immune profile of those who have recovered from SARS-CoV-2 have shown that there is still inflammation, vascular damage, and immune cell differentiation two to eight months after the infection. In particular, these patients appear to have increased levels of cytokines compared to healthy controls, and these cytokines are associated with the persistence of symptoms [13].

Moreover, this significant increase in cytokines, especially interleukin 6 (IL-6), which penetrates the blood–brain barrier (BBB), appears to alter neuronal functions and cause complications in the central nervous system (CNS), dysautonomia, depression, and hearing loss [14]. A recent genome-wide association study of long COVID-19 has revealed a significant association between a single nucleotide polymorphism located in the FOXP4 locus (chr6) and an increased risk of long COVID-19, implying that individuals may be genetically predisposed to its development [15]. However, the exact mechanism may result from the combination of various abovementioned mechanisms and depend on the affected system.

Here, we aim to review the leading hypotheses on the pathophysiology of long COVID-19. 

## 2. Cardiovascular System

The cardiovascular system is one of the most common systems involved in long COVID-19 syndrome. The most common symptoms are chest pain, fatigue, shortness of breath, and exercise intolerance, which can last for a long time after the infection [16]. Myocarditis, pericarditis, arrhythmias, and thromboembolic events have been reported as more severe clinical complications [17]. A retrospective cohort study evaluated the long-term cardiovascular outcomes in COVID-19 survivors among non-vaccinated people and showed that people who did not receive vaccines suffered from more cerebrovascular accidents (CVA). Strokes, arrhythmias, myocarditis, ischemic heart diseases, and thromboembolic disorders were some of them, leading to increased complications and adverse outcomes [18]. Similar results arose from a study including almost 155,000 patients recovered from COVID-19 and 5 million historical controls. After the first 30 days of the infection, cardiovascular disease was increased, regardless of the hospitalization status, age, sex, or comorbidities of the patient [17]. A prospective study from the United Kingdom was conducted on patients who experienced COVID-19 and had symptoms of long COVID-19. Quantitative magnetic resonance imaging (MRI) results supported that over half had persistent cardiac abnormalities within twelve months. Biomarkers like troponin and Brain Natriuretic Peptide (BNP) could not predict the findings [16].

Although the exact mechanisms that lead to the long-term cardiovascular symptoms of COVID-19 are not fully understood, plenty of proposed mechanisms are suggested in the literature. One of the most prevalent theories is based on endothelial dysfunction and microvascular injury, also known as endothelitis, caused by the prolonged inflammation reported in long COVID-19 [19,20,21]. Considering that other viruses can cause cardiovascular events as well, the possibility of increased and persistent inflammation causing similar events in long COVID-19 is plausible and likely [22]. Elevated levels of IL-6, 1-β, and tumor necrosis factor (TNF) can cause systemic manifestations and organ-specific problems, including in the heart. The combination of the inflammatory reaction with endothelial activation by the virus leads to thrombosis via the activation of the coagulation pathway [23]. The coagulation process is elicited in response to increased tissue factor (TF) secretion. In particular, thrombin is generated after the creation of the tenase complex, which consists of TF and factor VIIa [24,25]. The primary function of thrombin is the formation of insoluble fibrin from soluble fibrinogen [24,25]. Notably, patients with post-COVID-19 syndrome are characterized by fibrinoid clots that are accompanied by the hyperactivation of platelets. When microclots are formed in patients suffering from the long-term effects of COVID-19, several inflammatory mediators play a significant role in this process, like serum amyloid A (SAA), alpha 2-antiplasmin (α2AP), various fibrinogen chains, platelet factor 4 (PF4) and VWF, as revealed by proteomics [8,26]. The multi-organ complications of post-COVID-19 syndrome arise from the combination of unresolved coagulopathy during the acute phase of the disease and a disturbed fibrinolytic system [8,26,27].

Fogarty et al. measured endothelial cell activation markers in 55 patients at a median time of 68 days after the initial infection and found elevated levels of von Willebrand factor, factor VIII, and thrombomodulin [28]. A study found elevated levels of endothelial cells in COVID-19 survivors, which were related to high cytokine levels, implying that endothelitis can be the result of inflammation [29]. Endothelial activation and dysfunction are created through endothelial cell apoptosis, mainly via direct contact with the virus, platelet activation through cytokines, and leukocyte adhesion. All these together affect vascular homeostasis. Furthermore, binding the SARS-CoV-2 virus to angiotensin-converting enzyme 2 (ACE-2) receptors on the endothelium results in the release of angiotensin 2 and reduced nitric oxide (NO) production, causing damage to the endothelium through increased oxidative stress and mitochondrial dysfunction [13,30,31]. Finally, the importance of ACE-2 in the homeostasis of the Renin–Angiotensin–Aldosterone System (RAAS) is already known, and the viral binding through the viral spike protein can cause significant dysregulation and cardiovascular symptoms [32].

The exact role of chronic immune dysregulation in the pathophysiology of the cardiac symptoms of long COVID-19 has yet to be clarified. Still, it may play a role in multiorgan involvement, including the heart [33]. The recognition of cell damage by macrophages and epithelial cells triggers a robust immune response, leading to collateral damage due to the excessive infiltration of immune cells [13]. This damage can last longer and eventually leave residual damage to organs such as the heart. Studies have shown increased levels of TNF, IL-1β, 4, 6, 7, 8, 10, and 15 two months after the infection, and increased levels of IL-1β, six and TNF-α eight months later. This indicates continuous inflammation, vascular injury, and the differentiation of immune cells. Moreover, these elevated levels of cytokines have been correlated with the persistence of COVID-19 symptoms [34,35]. These findings are consistent with those of another study in which COVID-19 patients underwent heart MRIs six and twelve months after infection, with persistent myocardial inflammation shown in almost half of them [16].

Some of the cardiovascular long COVID-19 symptoms could be explained by the dysregulation in the autonomous nerve system caused by SARS-CoV-2. Dysautonomia in long COVID-19 can be caused by the pronounced release of cytokines, accompanied by the activation of the sympathetic system and the secretion of a large number of catecholamines. This promotes the higher secretion of more cytokines, creating a vicious cycle of detrimental events for the autonomous nervous system [36]. The second mechanism suspected to cause dysautonomia is mediated by autoantibodies, as it is well known that in COVID-19, a wide range of autoantibodies that may lead to the dysfunction of the autonomic nervous system are produced, causing tachycardia and a reduction in the vascular tone [37,38]. Peripheral nerve system (PNS) and CNS receptor dysfunctions induced by viral infections through antibodies have also been described. In particular, the production of autoantibodies against catecholamine, angiotensin 2, and endothelin receptors has been found to affect the heart rate [38]. In another case, small-fiber neuropathy has been implicated in post-COVID-19 syndrome and considered a cause of dysautonomia [39]. Consistent with the above, cases with dysregulated tissue oxygen supply, such as postural orthostatic tachycardia syndrome (POTS) and orthostatic hypotension (OH), can contribute to dysautonomia, owing to peripheral vasoconstriction [40].

## 3. Respiratory System

Pulmonary symptoms in long COVID-19 are common. A study from the USA of over 16,000 patients who suffered from the disease showed that as many as 40% experienced a persistent shortness of breath [41]. Other possible complications and symptoms are cough, pneumothorax, pulmonary hypertension, and infections [42,43,44]. However, the most severe manifestation of the pulmonary complications of long COVID-19 is pulmonary fibrosis (PF). Abnormal chest computerized tomographies (CTs) and pulmonary function tests (PFTs) have been reported even several months after the disease [45]. The potential predictors of pulmonary fibrosis are advanced age, comorbidities, the male sex, elevated d-dimers, and the elevation of specific inflammation markers four weeks after infection [45,46].

The pathophysiology of PF in long COVID-19 can be partially explained by the action of macrophages trying to repair alveolar damage and attracting fibroblasts. The combined activity of growth factors (GF), such as vascular endothelial GF and fibroblast GF, promotes the process of angiogenesis through the accumulation of endothelial cells. In long COVID-19, prolonged inflammation leads to prolonged fibroblast activity and permanent fibroblastic tissue [47]. It has also been reported that prolonged oxygen administration in the lungs during acute infection can increase oxidative stress, leading to pulmonary fibrosis [48]. Another implicated mechanism is the immune cell infiltration and increased cytokine production that leads to matrix metalloproteinases activating fibrotic adaptations in lung microcirculation [49].

Cases of shortness of breath, but without apparent lung disease during imaging or functional tests, have also been described. A possible mechanism for this is dysautonomia in either brain regions or intrathoracic receptors [38,50].

The respiratory long COVID-19 symptoms can also be explained by the vascular disorders it creates, leading to damage to the microcirculation of the lung and eventually pulmonary hypertension [51,52,53]. Various mechanisms of thrombus formation in the lung have been described. Clots may form in either small capillaries or large pulmonary arteries, and may also present as septic thromboembolic [54]. Endothelial cells come into contact with viral products and cause thrombosis. A dysfunctional endothelium combined with the hyperinflammation present in COVID-19 activates the coagulation pathways [55].

Persistent viral toxicity is another potential pathophysiologic mechanism involved in long COVID-19. For example, a high viral burden and the persistence of SARS-CoV-2 or the reactivation of Epstein–Barr virus (EBV) during acute infection have been regarded as significant risk factors for the long-term effects of COVID-19. Consistent with the above, the persistence of the infection caused either by EBV or SARS-CoV-2 can account for the sustained immune response, thus contributing to post-COVID-19 syndrome [56]. Alternatively, the activation of dormant viruses in terms of oxidative stress or immunosuppression can lead to the pathology of post-COVID-19 syndrome [57,58]. In parallel, researchers have used autoantibodies against type I interferons (IFNs) as predictive biomarkers for post-COVID-19 syndrome [59].

Spike protein binds with pneumonocytes type II, expressing elevated levels of ACE-2. In the case of pathological or delayed healing, long COVID-19 symptoms such as a shortness of breath and cough will be clinically observed. This pathological healing, along with the regeneration of respiratory cells, could explain the presentation of imaging and functional test abnormalities over time [49]. Persistent inflammation in the lung has also been shown by a study that performed positron emission tomography–CT (PET–CT) in patients who needed mechanical ventilation in the acute phase of the infection and complained of chronic respiratory symptoms [60].

Finally, another mechanism proposed to be implicated in the presentation of a chronic cough in long COVID-19 may involve chronic neuroinflammation and the stimulation of the vagal sensory nerves [61].

## 4. Central Nervous System

CNS symptoms are frequent, accounting for approximately 22% of long COVID-19 manifestations [62]. The most common ones are difficulties relating to concentration, brain fog, insomnia, anosmia, headaches, and amnesia [63]. More severe manifestations include encephalopathy, stroke, and seizures [64]. These symptoms can persist even one year after the infection [65]. The potential risk factors for neurological manifestations of long COVID-19 are a history of hospitalization and female gender. In contrast, age remains a controversial factor since some studies have found an association and others have not [66,67,68,69]. Another study correlated neurological symptoms with the presence of respiratory ones [70].

SARS-CoV-2 can invade the CNS in various ways. The first is through the nasal cavity, where the virus connects with the ACE-2 receptors of the olfactory epithelium, invades its nerves, and eventually reaches the brainstem through the pathway of the olfactory tract, as indicated by an autopsy study of 33 patients with COVID-19 in which ACE2 was detected in olfactory mucosa [71]. On the other hand, although researchers have been cautious regarding the question of whether the virus can enter the CNS, data from a study of the autopsies of 44 COVID-19 patients and an observational study with quantified viral RNA in plasma samples of COVID-19 patients support the theory that, through a viremic phase, the virus spreads via a hematogenous route throughout the body, including the brain; the BBB can become vulnerable in cases of infection, and thus the virus can invade the brain more easily [72,73,74]. Finally, there are reports that it may reach the CNS via the gastrointestinal tract since the virus is present in enterocytes, and that it can reach the CNS via the vagus nerve, as indicated by a prospective study that examined human gut samples of COVID-19 patients [75].

When the virus enters the CNS, it stimulates neuroinflammation. Therefore, microglia and astrocytes are activated, as in many neurodegenerative diseases, and this could explain several of the neurological symptoms of long COVID-19 [76]. This theory is also supported by two studies in which a brain biopsy was performed, showing inflammation and the activation of neutrophils and macrophages [73,77].

The area of the brain most easily affected by infections is the hippocampus, the dysfunction of which can be linked to cases of memory loss [78]. There are even reports in the literature of structural changes in the hippocampus in MRIs of long COVID-19 patients; these changes were associated with memory and smell loss [79]. In addition, a study using PET showed that specific brain regions develop hypometabolism, including the hippocampus and brainstem [80]. These metabolic disorders probably develop in the context of immunological disease, as the cerebrospinal fluid (CSF) of these patients was normal upon examination, and they improved with the administration of corticosteroids and immunoglobulin [81]. A second potential mechanism implicated in hypometabolism could involve mitochondrial dysfunction, since the virus uses mitochondria to replicate and, in combination with the inflammatory state, reduces the energy capacities of the cells [82].

Prolonged inflammation in the brain was shown by a study that used neuropsychiatric and neurophysiological tests and found central neuromuscular fatigue, apathy, and executive dysfunction in long COVID-19 patients. The primary mechanism is thought to involve the alteration of neuronal function, mainly via a significant increase in cytokines, especially IL-6, which penetrates the BBB and causes complications in the CNS [83,84]. Increased IL-6 also appears to decrease the expression of gamma-aminobutyric acid (GABA) receptors, which can lead to neuromuscular fatigue [85]. IL-6 overproduction is also associated with depressive symptoms, and its increased concentration is an independent risk factor. The normalization of IL-6 relieves the symptoms of depression [86].

Except for IL-6, an overproduction of IL-4 has also been observed; this causes continuous neuroinflammation and is mainly involved in memory symptoms. Persistent neuroinflammation caused by IL-4 can lead to a change in neuronal-enriched extracellular vesicle (nEV) proteins [87]. Elevated levels of TGF-β and IL-8 have been observed in patients with brain fog, and high levels of neuronal dysfunction biomarkers such as amyloid-beta, neurogranin, total tau, and pT181-tau have been observed in patients with persistent symptoms [87].

In addition, increased levels of IL-1, 6, and TNF-α may cause stress to the cochlear cells and, in combination with direct infiltration by the virus, lead to irreversible hearing loss [88].

Immune dysregulation is another pathophysiological mechanism involved in the spectrum of nervous system long COVID-19 pathophysiology. Elevated antiganglioside antibodies, which could be produced by cross-reactivity with the virus, have been found in patients with PNS symptoms such as Guillen–Barre and encephalomyelitis [89]. Patients have also been described to have cerebral infarct and positive anticardiolipin, anti-β2 microglobulin antibodies, and lupus anticoagulant. However, the positivity of these antibodies may be false due to inflammation and could have no clinical impact [90,91].

Finally, another contributing factor is microthrombosis in the brain, either from mitochondrial dysfunction or hypercoagulopathy. Several mechanisms have been described to induce microthrombi in the brain [92]. Post-mortem evidence of thrombotic microangiopathy and endothelitis has been found in some patients [93]. MRI studies show either cerebral infarction or microvascular damage [94]. Furthermore, increased levels of cytokines such as IL-8 and TNF lead to an increase in von Willebrand factor and, therefore, in platelet aggregation thrombosis [95].

The loss of taste and smell [96,97,98], as well as brain fog with difficulty concentrating and memory loss, have been reported [99,100]. In cohorts of patients with six-month follow-ups, many of them reported persistent symptoms of depression, anxiety, insomnia, and post-traumatic stress disorder [101,102]. One possible mechanism is the direct entry of the virus into neurons. An autopsy study showed changes in brain parenchyma caused by inflammation [103,104]. Due to an increase in the permeability of the BBB caused by cytokines, microorganisms and proinflammatory molecules enter the CNS, which affect the functions of the limbic system, leading to depression [105]. This is further strengthened by the activation of microglia via inflammation [106]. A second theory is that the virus-induced damage to sensory neurons leads to reduced CSF outflow and the congestion of the lymphatic system, resulting in the accumulation of toxins in the CNS [107]. Markers of brain damage, such as the neurofilament light chain in peripheral blood, have also been found [108].

## 5. Immune System

In its attempt to produce antibodies against the virus and its structures, the human body produces antibodies against its own structures due to the viral proteins mimicking human proteins [57,109]. The production of anti-ACE-2 autoantibodies has been described in several patients. Although their role is not fully understood, they could theoretically affect the action of ACE-2 by controlling hypertension and the functions of the renin–angiotensin system [110,111]. 

Other autoantibodies that have been detected target interferons [112], thus affecting the immune response and causing viral persistence, as well as antibodies that cause Guillain–Barre syndrome, thrombocytopenia, and systemic lupus erythematosus [113,114,115]. For instance, Cervia and colleagues highlighted the presence of a SARS-CoV-2-specific immunoglobulin signature driving post-COVID-19 syndrome [116]. In particular, low IgM and IgG3 titers can increase individuals’ susceptibility to the long-term effects of COVID-19, accompanied by the diminished secretion of type I IFNs [116].

There are reports of patients with COVID-19 developing macrophage activation syndrome. Measured tryptase levels have been found to be significantly elevated compared to healthy controls and correlated with IL-6 levels [117,118].

The hyperactivation of the immune system causes the production of a wide range of autoantibodies specific to G-protein-coupled receptors (GPCR), cytokines, or tissues and cell structures in the acute and chronic phases [37,119]. This leads to the inhibition of catecholamine and acetylcholine signaling, resulting in the dysfunction of the autonomous nervous system [38]. In one case report, a DNA aptamer was given, and this led to the neutralization of the anti-GPCR autoantibodies, improved retinal microcirculation, and the elimination of long COVID-19 symptoms [120]. 

These autoantibodies can also trigger other autoimmune diseases such as rheumatoid arthritis and fibromyalgia [121]. In patients with long COVID-19, there is an accumulation of autoantibodies in their circulation, contributing to the emergence of connective tissue disorders, in which connective tissue and muscle are disrupted due to the presence of autoantibodies [122]. Such disorders include the following: myositis, lupus, and arthritis [122].

SARS-CoV-2-induced immune dysregulation also causes the prolonged activation of T-cells and the expression of exhaustion markers, such as PD1 and TIM-3 in CD4+ and CD8+ even eight months after the infection [123,124]. This leads to a decrease in the production of cytokines, a dysfunction in the production of memory cells [125,126], and the expression of immunoinhibitory receptors in lymphoid and myeloid cells [127]. Finally, there is a decrease in the absolute number of lymphocytes and dendritic cells [128,129]. In severe forms of COVID-19, the elimination of B and T lymphocytes is linked to the chronic inflammation seen in post-COVID-19 syndrome. 

## 6. Other Systems

Kidney involvement in long COVID-19 is not rare. Studies have shown that in patients with long COVID-19, the impairment of kidney function that occurs in the acute phase can last up to 3 months [130]. The most likely causes in the literature are hemodynamic instability, coagulation disorders, and systemic inflammation [131,132]. Furthermore, the virus itself can enter renal cells through ACE-2, causing glomerulopathy that can manifest as proteinuria, hematuria, and renal failure, especially in patients with risk factors [133,134,135,136].

Diabetes mellitus and its dysregulation is an apparent complication of COVID-19 and long COVID-19. Two months after the initial infection, in patients with previously normal glucose levels, hyperglycemia and insulin resistance can be found relatively frequently [137]. The virus enters the pancreatic cells directly through ACE-2 receptors and destroys the pancreatic cells; consequently, diabetes mellitus can develop [138]. This can be further aggravated by the use of corticosteroids to treat COVID-19 [139]. In a person with pre-existing diabetes mellitus, glycemic control may worsen as the virus infects the islets of the pancreas and reduces insulin secretion [140]. There continue to be reports of dysregulated thyroid and parathyroid function, as well as deficient levels of vitamin D in patients with long COVID-19 [141,142,143]. Bone health can be affected by long COVID-19 as the virus directly affects the function of osteoblasts and osteoclasts. In particular, it inhibits osteoblasts and enhances the activity of osteoclasts [144]. Furthermore, cytokine storms can cause bone loss in animal models and, in combination with corticosteroid therapy and low vitamin D, may worsen bone health [145].

The virus can persist in the gut of patients for a long time. This may not be directly related to the symptoms of long COVID-19, but in some cases, it could explain some of them [146]. The virus enters the gastrointestinal system through ACE-2 receptors, which are present on enterocytes and hepatocytes, and thus cause abnormal liver function and gastrointestinal symptoms, such as nausea, dysphagia, abdominal pain, and irritable bowel syndrome [147,148]. These symptoms may persist up to two months after infection in a percentage of patients [149]. Four months after the infection, the nucleocapsid proteins of the virus have been detected in intestinal biopsies of the feces and in their viral RNA, even in patients with a negative nasopharyngeal PCR [146,150]. It also appears to cause a change in the intestinal microbiome that persists for up to six months, causing dysbiosis significantly associated with long COVID-19 [151].

In the liver, the virus behaves like other hepatotropic viruses [152]. It has been detected in hepatocytes during biopsies of patients with long COVID-19 [152]. Other possible mechanisms, besides the persistence of the virus in the liver, are the chronic inflammation and accumulation of cytokines, leading to abnormal liver function [153], as well as changes in the microbiome that cause dysbiosis and expose the liver to the altered microflora that cause reactive inflammation [154,155]. Finally, there are reports of cases of cholangiopathy after COVID-19 infection, although the exact mechanism is not understood yet [156].

Patients with long COVID-19 often complain of a syndrome of chronic malaise, myalgias, depressive symptoms, and sleep disorders [157]. Finally, social factors, such as the isolation, fear, and uncertainty experienced by the world during this period, may have led to symptoms of anxiety, depression, memory loss, and behavioral disorders [158,159].

**Figure 1:**
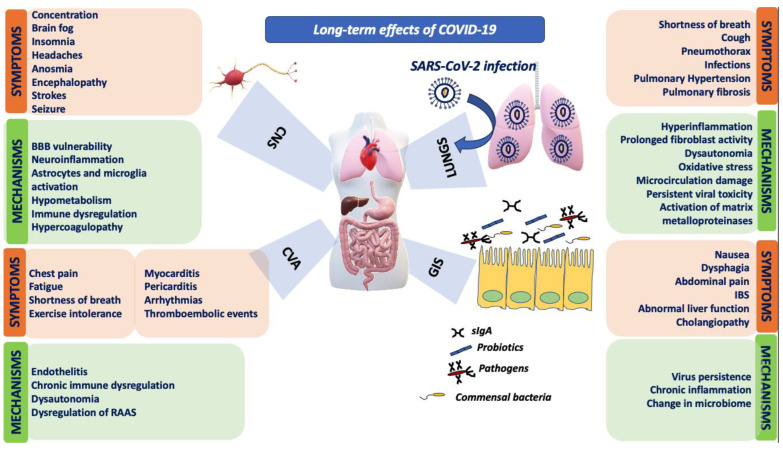
Mechanisms and clinical manifestations of long COVID-19 in various systems. BBB: blood–brain barrier, CNS: central nervous system, COVID-19: coronavirus disease 2019, CVA: cardiovascular system, GIS: gastrointestinal system; IBS: irritable bowel syndrome, RAAS: renin–angiotensin–aldosterone system, SARS-CoV-2: severe acute respiratory syndrome 2, sIgA: soluble immunoglobulin A.

Figure 1 summarizes the clinical manifestations and the underlying mechanisms of long COVID-19.

## 7. Conclusions

This manuscript provides a comprehensive overview of the pathophysiology of long COVID-19, shedding light on this persistent condition’s complex and multifaceted nature. The prolonged persistence of symptoms, spanning weeks to months after the initial infection, underscores the need for a deeper understanding of the pathophysiological processes at play. Moreover, understanding the underlying mechanisms of this disease may pave the way for improved diagnostics, management, and targeted therapeutic interventions.

Emerging evidence suggests that long COVID-19 is not solely an extension of the acute infection, but a unique entity with distinct mechanisms. Chronic inflammation, the dysregulation of the immune response, and persistent viral reservoirs have been implicated in the perpetuation of symptoms. Additionally, vascular endothelial dysfunction, autonomic dysregulation, and neuronal abnormalities contribute to these patients’ diverse clinical presentations. Genetic susceptibility, the host immune response, and viral factors likely dictate the severity and duration of long COVID-19. Some of the pathophysiological mechanisms seem to be more established than others due to prospective studies supporting them. 

On the other hand, many of the mechanisms and hypotheses proposed by experts need further investigation to establish them. Endothelitis, hypercoaguolopathy, prolonged inflammation, and immune dysregulation are the most studied and established mechanisms. In contrast, the dysregulation of RAAS, dysautonomia, and oxidative stress need further investigation in order to understand and establish their role in the pathogenesis of long COVID-19. Table S1 summarizes most of the studies included in this review, highlighting which mechanisms have been confirmed to a greater extent by prospective studies and which represent proposed mechanisms and expert opinions. These complex interactions warrant further exploration, mainly due to the long-term implications on public health and healthcare systems worldwide. Moreover, the vast heterogeneity observed in long COVID-19 calls for a personalized approach to diagnosis and management. Identifying the biomarkers associated with disease progression and recovery may facilitate risk stratification and guide tailored therapeutic strategies for patients grappling with this condition.

While significant progress has been made in deciphering the pathophysiological underpinnings of long COVID-19, many unexplored facets still warrant further investigation. Longitudinal studies, robust clinical trials, and interdisciplinary collaborations will be instrumental in advancing our understanding of this enigmatic condition.

## Data Availability

The data presented in this study are available upon request from the corresponding author.

## Supplementary Materials

The following supporting information can be downloaded at: https://www.mdpi.com/article/10.3390/microorganisms11102458/s1, Table S1: Summary of the most established mechanisms for the symptoms of long COVID-19 as supported by published evidence.

## References

[1] Robertson M.M., Qasmieh S.A., Kulkarni S.G., Teasdale C.A., Jones H.E., McNairy M., Borrell L.N., Nash D. (2023). The Epidemiology of Long Coronavirus Disease in US Adults. Clin. Infect. Dis. Off. Publ. Infect. Dis. Soc. Am..

[2] Department of Health and Human Services, Office of the Assistant Secretary for Health (2022). National Research Action Plan on Long COVID.

[3] Greenhalgh T., Knight M., A’Court C., Buxton M., Husain L. (2020). Management of Post-Acute COVID-19 in Primary Care. BMJ.

[4] Raveendran A.V. (2021). Long COVID-19: Challenges in the Diagnosis and Proposed Diagnostic Criteria. Diabetes Metab. Syndr. Clin. Res. Rev..

[5] Davis H.E., McCorkell L., Vogel J.M., Topol E.J. (2023). Long COVID: Major Findings, Mechanisms and Recommendations. Nat. Rev. Microbiol..

[6] Cutler D.M., Summers L.H. (2020). The COVID-19 Pandemic and the $16 Trillion Virus. JAMA.

[7] Raveendran A.V., Jayadevan R., Sashidharan S. (2021). Long COVID: An Overview. Diabetes Metab. Syndr..

[8] Pretorius E., Venter C., Laubscher G.J., Kotze M.J., Oladejo S.O., Watson L.R., Rajaratnam K., Watson B.W., Kell D.B. (2022). Prevalence of Symptoms, Comorbidities, Fibrin Amyloid Microclots and Platelet Pathology in Individuals with Long COVID/Post-Acute Sequelae of COVID-19 (PASC). Cardiovasc. Diabetol..

[9] Carfì A., Bernabei R., Landi F. (2020). Persistent Symptoms in Patients After Acute COVID-19. JAMA.

[10] Twomey R., DeMars J., Franklin K., Culos-Reed S.N., Weatherald J., Wrightson J.G. (2022). Chronic Fatigue and Postexertional Malaise in People Living With Long COVID: An Observational Study. Phys. Ther..

[11] Subramanian A., Nirantharakumar K., Hughes S., Myles P., Williams T., Gokhale K.M., Taverner T., Chandan J.S., Brown K., Simms-Williams N. (2022). Symptoms and Risk Factors for Long COVID in Non-Hospitalized Adults. Nat. Med..

[12] Tsampasian V., Elghazaly H., Chattopadhyay R., Debski M., Naing T.K.P., Garg P., Clark A., Ntatsaki E., Vassiliou V.S. (2023). Risk Factors Associated With Post−COVID-19 Condition: A Systematic Review and Meta-Analysis. JAMA Intern. Med..

[13] Haunhorst S., Bloch W., Wagner H., Ellert C., Krüger K., Vilser D.C., Finke K., Reuken P., Pletz M.W., Stallmach A. (2022). Long COVID: A Narrative Review of the Clinical Aftermaths of COVID-19 with a Focus on the Putative Pathophysiology and Aspects of Physical Activity. Oxf. Open Immunol..

[14] Maltezou H.C., Pavli A., Tsakris A. (2021). Post-COVID Syndrome: An Insight on Its Pathogenesis. Vaccines.

[15] Lammi V., Nakanishi T., Jones S.E., Andrews S.J., Karjalainen J., Cortés B., O’Brien H.E., Fulton-Howard B.E., Haapaniemi H.H., Schmidt A. (2023). Genome-Wide Association Study of Long COVID. medRxiv.

[16] Roca-Fernandez A., Wamil M., Telford A., Carapella V., Borlotti A., Monteiro D., Thomaides-Brears H., Kelly M., Dennis A., Banerjee R. (2023). Cardiac Abnormalities in Long COVID 1-Year Post-SARS-CoV-2 Infection. Open Heart.

[17] Xie Y., Xu E., Bowe B., Al-Aly Z. (2022). Long-Term Cardiovascular Outcomes of COVID-19. Nat. Med..

[18] Wang W., Wang C.-Y., Wang S.-I., Wei J.C.-C. (2022). Long-Term Cardiovascular Outcomes in COVID-19 Survivors among Non-Vaccinated Population: A Retrospective Cohort Study from the TriNetX US Collaborative Networks. eClinicalMedicine.

[19] Evans P.C., Rainger G.E., Mason J.C., Guzik T.J., Osto E., Stamataki Z., Neil D., Hoefer I.E., Fragiadaki M., Waltenberger J. (2020). Endothelial Dysfunction in COVID-19: A Position Paper of the ESC Working Group for Atherosclerosis and Vascular Biology, and the ESC Council of Basic Cardiovascular Science. Cardiovasc. Res..

[20] Castro P., Palomo M., Moreno-Castaño A.B., Fernández S., Torramadé-Moix S., Pascual G., Martinez-Sanchez J., Richardson E., Téllez A., Nicolas J.M. (2022). Is the Endothelium the Missing Link in the Pathophysiology and Treatment of COVID-19 Complications?. Cardiovasc. Drugs Ther..

[21] Lambadiari V., Mitrakou A., Kountouri A., Thymis J., Katogiannis K., Korakas E., Varlamos C., Andreadou I., Tsoumani M., Triantafyllidi H. (2021). Association of COVID-19 with Impaired Endothelial Glycocalyx, Vascular Function and Myocardial Deformation 4 Months after Infection. Eur. J. Heart Fail..

[22] Corrales-Medina V.F., Alvarez K.N., Weissfeld L.A., Angus D.C., Chirinos J.A., Chang C.-C.H., Newman A., Loehr L., Folsom A.R., Elkind M.S. (2015). Association between Hospitalization for Pneumonia and Subsequent Risk of Cardiovascular Disease. JAMA.

[23] Batiha G.E.-S., Al-Kuraishy H.M., Al-Gareeb A.I., Welson N.N. (2022). Pathophysiology of Post-COVID Syndromes: A New Perspective. Virol. J..

[24] Szotowski B., Antoniak S., Poller W., Schultheiss H.-P., Rauch U. (2005). Procoagulant Soluble Tissue Factor Is Released from Endothelial Cells in Response to Inflammatory Cytokines. Circ. Res..

[25] Esmon C.T. (2005). The Interactions between Inflammation and Coagulation. Br. J. Haematol..

[26] Kruger A., Vlok M., Turner S., Venter C., Laubscher G.J., Kell D.B., Pretorius E. (2022). Proteomics of Fibrin Amyloid Microclots in Long COVID/Post-Acute Sequelae of COVID-19 (PASC) Shows Many Entrapped pro-Inflammatory Molecules That May Also Contribute to a Failed Fibrinolytic System. Cardiovasc. Diabetol..

[27] Østergaard L. (2021). SARS CoV-2 Related Microvascular Damage and Symptoms during and after COVID-19: Consequences of Capillary Transit-time Changes, Tissue Hypoxia and Inflammation. Physiol. Rep..

[28] Fogarty H., Townsend L., Morrin H., Ahmad A., Comerford C., Karampini E., Englert H., Byrne M., Bergin C., O’Sullivan J.M. (2021). Persistent Endotheliopathy in the Pathogenesis of Long COVID Syndrome. J. Thromb. Haemost. JTH.

[29] Chioh F.W., Fong S.-W., Young B.E., Wu K.-X., Siau A., Krishnan S., Chan Y.-H., Carissimo G., Teo L.L., Gao F. (2021). Convalescent COVID-19 Patients Are Susceptible to Endothelial Dysfunction Due to Persistent Immune Activation. eLife.

[30] Vassiliou A.G., Vrettou C.S., Keskinidou C., Dimopoulou I., Kotanidou A., Orfanos S.E. (2023). Endotheliopathy in Acute COVID-19 and Long COVID. Int. J. Mol. Sci..

[31] Ahamed J., Laurence J. (2022). Long COVID Endotheliopathy: Hypothesized Mechanisms and Potential Therapeutic Approaches. J. Clin. Investig..

[32] Cooper S.L., Boyle E., Jefferson S.R., Heslop C.R.A., Mohan P., Mohanraj G.G.J., Sidow H.A., Tan R.C.P., Hill S.J., Woolard J. (2021). Role of the Renin-Angiotensin-Aldosterone and Kinin-Kallikrein Systems in the Cardiovascular Complications of COVID-19 and Long COVID. Int. J. Mol. Sci..

[33] Gyöngyösi M., Alcaide P., Asselbergs F.W., Brundel B.J.J.M., Camici G.G., Martins P.D.C., Ferdinandy P., Fontana M., Girao H., Gnecchi M. (2023). Long COVID and the Cardiovascular System—Elucidating Causes and Cellular Mechanisms in Order to Develop Targeted Diagnostic and Therapeutic Strategies: A Joint Scientific Statement of the ESC Working Groups on Cellular Biology of the Heart and Myocardial and Pericardial Diseases. Cardiovasc. Res..

[34] Peluso M.J., Deitchman A.N., Torres L., Iyer N.S., Munter S.E., Nixon C.C., Donatelli J., Thanh C., Takahashi S., Hakim J. (2021). Long-Term SARS-CoV-2-Specific Immune and Inflammatory Responses in Individuals Recovering from COVID-19 with and without Post-Acute Symptoms. Cell Rep..

[35] Schultheiß C., Willscher E., Paschold L., Gottschick C., Klee B., Henkes S.-S., Bosurgi L., Dutzmann J., Sedding D., Frese T. (2022). The IL-1β, IL-6, and TNF Cytokine Triad Is Associated with Post-Acute Sequelae of COVID-19. Cell Rep. Med..

[36] Low R.N., Low R.J., Akrami A. (2020). A Cytokine-Based Model for the Pathophysiology of Long COVID Symptoms.

[37] Wallukat G., Hohberger B., Wenzel K., Fürst J., Schulze-Rothe S., Wallukat A., Hönicke A.-S., Müller J. (2021). Functional Autoantibodies against G-Protein Coupled Receptors in Patients with Persistent Long-COVID-19 Symptoms. J. Transl. Autoimmun..

[38] Dani M., Dirksen A., Taraborrelli P., Torocastro M., Panagopoulos D., Sutton R., Lim P.B. (2021). Autonomic Dysfunction in “Long COVID”: Rationale, Physiology and Management Strategies. Clin. Med. Lond. Engl..

[39] Abrams R.M.C., Simpson D.M., Navis A., Jette N., Zhou L., Shin S.C. (2022). Small Fiber Neuropathy Associated with SARS-CoV-2 Infection. Muscle Nerve.

[40] Vernino S., Bourne K.M., Stiles L.E., Grubb B.P., Fedorowski A., Stewart J.M., Arnold A.C., Pace L.A., Axelsson J., Boris J.R. (2021). Postural Orthostatic Tachycardia Syndrome (POTS): State of the Science and Clinical Care from a 2019 National Institutes of Health Expert Consensus Meeting—Part 1. Auton. Neurosci..

[41] Perlis R.H., Santillana M., Ognyanova K., Safarpour A., Lunz Trujillo K., Simonson M.D., Green J., Quintana A., Druckman J., Baum M.A. (2022). Prevalence and Correlates of Long COVID Symptoms among US Adults. JAMA Netw. Open.

[42] Jacobs L.G., Gourna Paleoudis E., Lesky-Di Bari D., Nyirenda T., Friedman T., Gupta A., Rasouli L., Zetkulic M., Balani B., Ogedegbe C. (2020). Persistence of Symptoms and Quality of Life at 35 Days after Hospitalization for COVID-19 Infection. PLoS ONE.

[43] Xiong Q., Xu M., Li J., Liu Y., Zhang J., Xu Y., Dong W. (2021). Clinical Sequelae of COVID-19 Survivors in Wuhan, China: A Single-Centre Longitudinal Study. Clin. Microbiol. Infect..

[44] Cortinovis M., Perico N., Remuzzi G. (2021). Long-Term Follow-up of Recovered Patients with COVID-19. Lancet.

[45] Zhao Y., Shang Y., Song W., Li Q., Xie H., Xu Q., Jia J., Li L., Mao H., Zhou X. (2020). Follow-up Study of the Pulmonary Function and Related Physiological Characteristics of COVID-19 Survivors Three Months after Recovery. EClinicalMedicine.

[46] Huang W., Wu Q., Chen Z., Xiong Z., Wang K., Tian J., Zhang S. (2021). The Potential Indicators for Pulmonary Fibrosis in Survivors of Severe COVID-19. J. Infect..

[47] Ojo A.S., Balogun S.A., Williams O.T., Ojo O.S. (2020). Pulmonary Fibrosis in COVID-19 Survivors: Predictive Factors and Risk Reduction Strategies. Pulm. Med..

[48] Tanni S.E., Fabro A.T., De Albuquerque A., Ferreira E.V.M., Verrastro C.G.Y., Sawamura M.V.Y., Ribeiro S.M., Baldi B.G. (2021). Pulmonary Fibrosis Secondary to COVID-19: A Narrative Review. Expert Rev. Respir. Med..

[49] Guizani I., Fourti N., Zidi W., Feki M., Allal-Elasmi M. (2021). SARS-CoV-2 and Pathological Matrix Remodeling Mediators. Inflamm. Res..

[50] Yong S.J. (2021). Persistent Brainstem Dysfunction in Long-COVID: A Hypothesis. ACS Chem. Neurosci..

[51] Dhawan R.T., Gopalan D., Howard L., Vicente A., Park M., Manalan K., Wallner I., Marsden P., Dave S., Branley H. (2021). Beyond the Clot: Perfusion Imaging of the Pulmonary Vasculature after COVID-19. Lancet Respir. Med..

[52] Ooi M.W.X., Rajai A., Patel R., Gerova N., Godhamgaonkar V., Liong S.Y. (2020). Pulmonary Thromboembolic Disease in COVID-19 Patients on CT Pulmonary Angiography—Prevalence, Pattern of Disease and Relationship to D-Dimer. Eur. J. Radiol..

[53] Van Kruijsdijk R.C., De Jong P.A., Abrahams A.C. (2020). Pulmonary Vein Thrombosis in COVID-19. BMJ Case Rep..

[54] Silva Andrade B., Siqueira S., de Assis Soares W.R., de Souza Rangel F., Santos N.O., Dos Santos Freitas A., Ribeiro da Silveira P., Tiwari S., Alzahrani K.J., Góes-Neto A. (2021). Long-COVID and Post-COVID Health Complications: An Up-to-Date Review on Clinical Conditions and Their Possible Molecular Mechanisms. Viruses.

[55] Astin R., Banerjee A., Baker M.R., Dani M., Ford E., Hull J.H., Lim P.B., McNarry M., Morten K., O’Sullivan O. (2023). Long COVID: Mechanisms, Risk Factors and Recovery. Exp. Physiol..

[56] Desimmie B.A., Raru Y.Y., Awadh H.M., He P., Teka S., Willenburg K.S. (2021). Insights into SARS-CoV-2 Persistence and Its Relevance. Viruses.

[57] Proal A.D., VanElzakker M.B. (2021). Long COVID or Post-Acute Sequelae of COVID-19 (PASC): An Overview of Biological Factors That May Contribute to Persistent Symptoms. Front. Microbiol..

[58] Rebman A.W., Aucott J.N. (2020). Post-Treatment Lyme Disease as a Model for Persistent Symptoms in Lyme Disease. Front. Med..

[59] Su Y., Yuan D., Chen D.G., Ng R.H., Wang K., Choi J., Li S., Hong S., Zhang R., Xie J. (2022). Multiple Early Factors Anticipate Post-Acute COVID-19 Sequelae. Cell.

[60] Bai Y., Xu J., Chen L., Fu C., Kang Y., Zhang W., Fakhri G.E., Gu J., Shao F., Wang M. (2021). Inflammatory Response in Lungs and Extrapulmonary Sites Detected by [18F] Fluorodeoxyglucose PET/CT in Convalescing COVID-19 Patients Tested Negative for Coronavirus. Eur. J. Nucl. Med. Mol. Imaging.

[61] Song W.-J., Hui C.K.M., Hull J.H., Birring S.S., McGarvey L., Mazzone S.B., Chung K.F. (2021). Confronting COVID-19-Associated Cough and the Post-COVID Syndrome: Role of Viral Neurotropism, Neuroinflammation, and Neuroimmune Responses. Lancet Respir. Med..

[62] Alkodaymi M.S., Omrani O.A., Fawzy N.A., Shaar B.A., Almamlouk R., Riaz M., Obeidat M., Obeidat Y., Gerberi D., Taha R.M. (2022). Prevalence of Post-Acute COVID-19 Syndrome Symptoms at Different Follow-up Periods: A Systematic Review and Meta-Analysis. Clin. Microbiol. Infect. Off. Publ. Eur. Soc. Clin. Microbiol. Infect. Dis..

[63] Premraj L., Kannapadi N.V., Briggs J., Seal S.M., Battaglini D., Fanning J., Suen J., Robba C., Fraser J., Cho S.-M. (2022). Mid and Long-Term Neurological and Neuropsychiatric Manifestations of Post-COVID-19 Syndrome: A Meta-Analysis. J. Neurol. Sci..

[64] García-Moncó J.C., Cabrera-Muras A., Collía-Fernández A., Erburu-Iriarte M., Rodrigo-Armenteros P., Oyarzun-Irazu I., Martínez-Condor D., Bilbao-González A., Carmona-Abellán M., Caballero-Romero I. (2020). Neurological Reasons for Consultation and Hospitalization during the COVID-19 Pandemic. Neurol. Sci. Off. J. Ital. Neurol. Soc. Ital. Soc. Clin. Neurophysiol..

[65] Stefanou M.-I., Palaiodimou L., Bakola E., Smyrnis N., Papadopoulou M., Paraskevas G.P., Rizos E., Boutati E., Grigoriadis N., Krogias C. (2022). Neurological Manifestations of Long-COVID Syndrome: A Narrative Review. Ther. Adv. Chronic Dis..

[66] Taquet M., Dercon Q., Luciano S., Geddes J.R., Husain M., Harrison P.J. (2021). Incidence, Co-Occurrence, and Evolution of Long-COVID Features: A 6-Month Retrospective Cohort Study of 273,618 Survivors of COVID-19. PLoS Med..

[67] Graham E.L., Clark J.R., Orban Z.S., Lim P.H., Szymanski A.L., Taylor C., DiBiase R.M., Jia D.T., Balabanov R., Ho S.U. (2021). Persistent Neurologic Symptoms and Cognitive Dysfunction in Non-Hospitalized COVID-19 “Long Haulers”. Ann. Clin. Transl. Neurol..

[68] Staffolani S., Iencinella V., Cimatti M., Tavio M. (2022). Long COVID-19 Syndrome as a Fourth Phase of SARS-CoV-2 Infection. Infez. Med..

[69] Parotto M., Myatra S.N., Munblit D., Elhazmi A., Ranzani O.T., Herridge M.S. (2021). Recovery after Prolonged ICU Treatment in Patients with COVID-19. Lancet Respir. Med..

[70] Whitaker M., Elliott J., Chadeau-Hyam M., Riley S., Darzi A., Cooke G., Ward H., Elliott P. (2021). Persistent Symptoms Following SARS-CoV-2 Infection in a Random Community Sample of 508,707 People. medRxiv.

[71] Meinhardt J., Radke J., Dittmayer C., Franz J., Thomas C., Mothes R., Laue M., Schneider J., Brünink S., Greuel S. (2021). Olfactory Transmucosal SARS-CoV-2 Invasion as a Port of Central Nervous System Entry in Individuals with COVID-19. Nat. Neurosci..

[72] Boldrini M., Canoll P.D., Klein R.S. (2021). How COVID-19 Affects the Brain. JAMA Psychiatry.

[73] Stein S.R., Ramelli S.C., Grazioli A., Chung J.-Y., Singh M., Yinda C.K., Winkler C.W., Sun J., Dickey J.M., Ylaya K. (2022). SARS-CoV-2 Infection and Persistence in the Human Body and Brain at Autopsy. Nature.

[74] Jacobs J.L., Bain W., Naqvi A., Staines B., Castanha P.M.S., Yang H., Boltz V.F., Barratt-Boyes S., Marques E.T.A., Mitchell S.L. (2022). Severe Acute Respiratory Syndrome Coronavirus 2 Viremia Is Associated With Coronavirus Disease 2019 Severity and Predicts Clinical Outcomes. Clin. Infect. Dis. Off. Publ. Infect. Dis. Soc. Am..

[75] Deffner F., Scharr M., Klingenstein S., Klingenstein M., Milazzo A., Scherer S., Wagner A., Hirt B., Mack A.F., Neckel P.H. (2020). Histological Evidence for the Enteric Nervous System and the Choroid Plexus as Alternative Routes of Neuroinvasion by SARS-CoV-2. Front. Neuroanat..

[76] Yang A.C., Kern F., Losada P.M., Agam M.R., Maat C.A., Schmartz G.P., Fehlmann T., Stein J.A., Schaum N., Lee D.P. (2021). Dysregulation of Brain and Choroid Plexus Cell Types in Severe COVID-19. Nature.

[77] DeMarino C., Lee M.-H., Cowen M., Steiner J.P., Inati S., Shah A.H., Zaghloul K.A., Nath A. (2023). Detection of SARS-CoV-2 Nucleocapsid and Microvascular Disease in the Brain: A Case Report. Neurology.

[78] Ritchie K., Chan D., Watermeyer T. (2020). The Cognitive Consequences of the COVID-19 Epidemic: Collateral Damage?. Brain Commun..

[79] Lu Y., Li X., Geng D., Mei N., Wu P.-Y., Huang C.-C., Jia T., Zhao Y., Wang D., Xiao A. (2020). Cerebral Micro-Structural Changes in COVID-19 Patients—An MRI-Based 3-Month Follow-up Study. EClinicalMedicine.

[80] Guedj E., Campion J.Y., Dudouet P., Kaphan E., Bregeon F., Tissot-Dupont H., Guis S., Barthelemy F., Habert P., Ceccaldi M. (2021). 18F-FDG Brain PET Hypometabolism in Patients with Long COVID. Eur. J. Nucl. Med. Mol. Imaging.

[81] Kas A., Soret M., Pyatigoskaya N., Habert M.-O., Hesters A., Le Guennec L., Paccoud O., Bombois S., Delorme C., on the behalf of CoCo-Neurosciences Study Group and COVID SMIT PSL Study Group (2021). The Cerebral Network of COVID-19-Related Encephalopathy: A Longitudinal Voxel-Based 18F-FDG-PET Study. Eur. J. Nucl. Med. Mol. Imaging.

[82] Stefano G.B., Büttiker P., Weissenberger S., Martin A., Ptacek R., Kream R.M. (2021). Editorial: The Pathogenesis of Long-Term Neuropsychiatric COVID-19 and the Role of Microglia, Mitochondria, and Persistent Neuroinflammation: A Hypothesis. Med. Sci. Monit. Int. Med. J. Exp. Clin. Res..

[83] Ortelli P., Ferrazzoli D., Sebastianelli L., Engl M., Romanello R., Nardone R., Bonini I., Koch G., Saltuari L., Quartarone A. (2021). Neuropsychological and Neurophysiological Correlates of Fatigue in Post-Acute Patients with Neurological Manifestations of COVID-19: Insights into a Challenging Symptom. J. Neurol. Sci..

[84] Trougakos I.P., Stamatelopoulos K., Terpos E., Tsitsilonis O.E., Aivalioti E., Paraskevis D., Kastritis E., Pavlakis G.N., Dimopoulos M.A. (2021). Insights to SARS-CoV-2 Life Cycle, Pathophysiology, and Rationalized Treatments That Target COVID-19 Clinical Complications. J. Biomed. Sci..

[85] Garcia-Oscos F., Salgado H., Hall S., Thomas F., Farmer G.E., Bermeo J., Galindo L.C., Ramirez R.D., D’Mello S., Rose-John S. (2012). The Stress-Induced Cytokine Interleukin-6 Decreases the Inhibition/Excitation Ratio in the Rat Temporal Cortex via Trans-Signaling. Biol. Psychiatry.

[86] Alpert O., Begun L., Garren P., Solhkhah R. (2020). Cytokine Storm Induced New Onset Depression in Patients with COVID-19. A New Look into the Association between Depression and Cytokines-Two Case Reports. Brain Behav. Immun.—Health.

[87] Sun B., Tang N., Peluso M.J., Iyer N.S., Torres L., Donatelli J.L., Munter S.E., Nixon C.C., Rutishauser R.L., Rodriguez-Barraquer I. (2021). Characterization and Biomarker Analyses of Post-COVID-19 Complications and Neurological Manifestations. Cells.

[88] Koumpa F.S., Forde C.T., Manjaly J.G. (2020). Sudden Irreversible Hearing Loss Post COVID-19. BMJ Case Rep..

[89] Dalakas M.C. (2020). Guillain-Barré Syndrome: The First Documented COVID-19–Triggered Autoimmune Neurologic Disease: More to Come with Myositis in the Offing. Neurol.—Neuroimmunol. Neuroinflamm..

[90] Bowles L., Platton S., Yartey N., Dave M., Lee K., Hart D.P., MacDonald V., Green L., Sivapalaratnam S., Pasi K.J. (2020). Lupus Anticoagulant and Abnormal Coagulation Tests in Patients with COVID-19. N. Engl. J. Med..

[91] Zhang Y., Cao W., Jiang W., Xiao M., Li Y., Tang N., Liu Z., Yan X., Zhao Y., Li T. (2020). Profile of Natural Anticoagulant, Coagulant Factor and Anti-Phospholipid Antibody in Critically Ill COVID-19 Patients. J. Thromb. Thrombolysis.

[92] Lee M.-H., Perl D.P., Nair G., Li W., Maric D., Murray H., Dodd S.J., Koretsky A.P., Watts J.A., Cheung V. (2021). Microvascular Injury in the Brains of Patients with COVID-19. N. Engl. J. Med..

[93] Hernández-Fernández F., Sandoval Valencia H., Barbella-Aponte R.A., Collado-Jiménez R., Ayo-Martín Ó., Barrena C., Molina-Nuevo J.D., García-García J., Lozano-Setién E., Alcahut-Rodriguez C. (2020). Cerebrovascular Disease in Patients with COVID-19: Neuroimaging, Histological and Clinical Description. Brain J. Neurol..

[94] Gulko E., Oleksk M.L., Gomes W., Ali S., Mehta H., Overby P., Al-Mufti F., Rozenshtein A. (2020). MRI Brain Findings in 126 Patients with COVID-19: Initial Observations from a Descriptive Literature Review. AJNR Am. J. Neuroradiol..

[95] Goshua G., Pine A.B., Meizlish M.L., Chang C.-H., Zhang H., Bahel P., Baluha A., Bar N., Bona R.D., Burns A.J. (2020). Endotheliopathy in COVID-19-Associated Coagulopathy: Evidence from a Single-Centre, Cross-Sectional Study. Lancet Haematol..

[96] Chopra V., Flanders S.A., O’Malley M., Malani A.N., Prescott H.C. (2021). Sixty-Day Outcomes Among Patients Hospitalized With COVID-19. Ann. Intern. Med..

[97] Arnold D.T., Hamilton F.W., Milne A., Morley A.J., Viner J., Attwood M., Noel A., Gunning S., Hatrick J., Hamilton S. (2021). Patient Outcomes after Hospitalisation with COVID-19 and Implications for Follow-up: Results from a Prospective UK Cohort. Thorax.

[98] Garrigues E., Janvier P., Kherabi Y., Le Bot A., Hamon A., Gouze H., Doucet L., Berkani S., Oliosi E., Mallart E. (2020). Post-Discharge Persistent Symptoms and Health-Related Quality of Life after Hospitalization for COVID-19. J. Infect..

[99] Heneka M.T., Golenbock D., Latz E., Morgan D., Brown R. (2020). Immediate and Long-Term Consequences of COVID-19 Infections for the Development of Neurological Disease. Alzheimers Res. Ther..

[100] Kaseda E.T., Levine A.J. (2020). Post-Traumatic Stress Disorder: A Differential Diagnostic Consideration for COVID-19 Survivors. Clin. Neuropsychol..

[101] Mazza M.G., De Lorenzo R., Conte C., Poletti S., Vai B., Bollettini I., Melloni E.M.T., Furlan R., Ciceri F., Rovere-Querini P. (2020). Anxiety and Depression in COVID-19 Survivors: Role of Inflammatory and Clinical Predictors. Brain. Behav. Immun..

[102] Rogers J.P., Chesney E., Oliver D., Pollak T.A., McGuire P., Fusar-Poli P., Zandi M.S., Lewis G., David A.S. (2020). Psychiatric and Neuropsychiatric Presentations Associated with Severe Coronavirus Infections: A Systematic Review and Meta-Analysis with Comparison to the COVID-19 Pandemic. Lancet Psychiatry.

[103] Romero-Sánchez C.M., Díaz-Maroto I., Fernández-Díaz E., Sánchez-Larsen Á., Layos-Romero A., García-García J., González E., Redondo-Peñas I., Perona-Moratalla A.B., Del Valle-Pérez J.A. (2020). Neurologic Manifestations in Hospitalized Patients with COVID-19: The ALBACOVID Registry. Neurology.

[104] Reichard R.R., Kashani K.B., Boire N.A., Constantopoulos E., Guo Y., Lucchinetti C.F. (2020). Neuropathology of COVID-19: A Spectrum of Vascular and Acute Disseminated Encephalomyelitis (ADEM)-like Pathology. Acta Neuropathol..

[105] Halaris A. (2019). Inflammation and Depression but Where Does the Inflammation Come From?. Curr. Opin. Psychiatry.

[106] Enache D., Pariante C.M., Mondelli V. (2019). Markers of Central Inflammation in Major Depressive Disorder: A Systematic Review and Meta-Analysis of Studies Examining Cerebrospinal Fluid, Positron Emission Tomography and Post-Mortem Brain Tissue. Brain. Behav. Immun..

[107] Wostyn P. (2021). COVID-19 and Chronic Fatigue Syndrome: Is the Worst yet to Come?. Med. Hypotheses.

[108] Ameres M., Brandstetter S., Toncheva A.A., Kabesch M., Leppert D., Kuhle J., Wellmann S. (2020). Association of Neuronal Injury Blood Marker Neurofilament Light Chain with Mild-to-Moderate COVID-19. J. Neurol..

[109] Dotan A., Muller S., Kanduc D., David P., Halpert G., Shoenfeld Y. (2021). The SARS-CoV-2 as an Instrumental Trigger of Autoimmunity. Autoimmun. Rev..

[110] Arthur J.M., Forrest J.C., Boehme K.W., Kennedy J.L., Owens S., Herzog C., Liu J., Harville T.O. (2021). Development of ACE2 Autoantibodies after SARS-CoV-2 Infection. PLoS ONE.

[111] Verano-Braga T., Martins A.L.V., Motta-Santos D., Campagnole-Santos M.J., Santos R.A.S. (2020). ACE2 in the Renin–Angiotensin System. Clin. Sci..

[112] Koning R., Bastard P., Casanova J.-L., Brouwer M.C., Van De Beek D., Van Agtmael M., Algera A.G., Appelman B., Van Baarle F., with the Amsterdam U.M.C. COVID-19 Biobank Investigators (2021). Autoantibodies against Type I Interferons Are Associated with Multi-Organ Failure in COVID-19 Patients. Intensive Care Med..

[113] Bhattacharjee S., Banerjee M. (2020). Immune Thrombocytopenia Secondary to COVID-19: A Systematic Review. SN Compr. Clin. Med..

[114] Son K., Jamil R., Chowdhury A., Mukherjee M., Venegas C., Miyasaki K., Zhang K., Patel Z., Salter B., Yuen A.C.Y. (2022). Circulating Anti-Nuclear Autoantibodies in COVID-19 Survivors Predict Long-COVID Symptoms. Eur. Respir. J..

[115] Caress J.B., Castoro R.J., Simmons Z., Scelsa S.N., Lewis R.A., Ahlawat A., Narayanaswami P. (2020). COVID-19–associated Guillain-Barré Syndrome: The Early Pandemic Experience. Muscle Nerve.

[116] Cervia C., Zurbuchen Y., Taeschler P., Ballouz T., Menges D., Hasler S., Adamo S., Raeber M.E., Bächli E., Rudiger A. (2022). Immunoglobulin Signature Predicts Risk of Post-Acute COVID-19 Syndrome. Nat. Commun..

[117] Weinstock L.B., Brook J.B., Walters A.S., Goris A., Afrin L.B., Molderings G.J. (2021). Mast Cell Activation Symptoms Are Prevalent in Long-COVID. Int. J. Infect. Dis. IJID Off. Publ. Int. Soc. Infect. Dis..

[118] Wechsler J.B., Butuci M., Wong A., Kamboj A.P., Youngblood B.A. (2022). Mast Cell Activation Is Associated with Post-Acute COVID-19 Syndrome. Allergy.

[119] Richter A.G., Shields A.M., Karim A., Birch D., Faustini S.E., Steadman L., Ward K., Plant T., Reynolds G., Veenith T. (2021). Establishing the Prevalence of Common Tissue-Specific Autoantibodies Following Severe Acute Respiratory Syndrome Coronavirus 2 Infection. Clin. Exp. Immunol..

[120] Hohberger B., Harrer T., Mardin C., Kruse F., Hoffmanns J., Rogge L., Heltmann F., Moritz M., Szewczykowski C., Schottenhamml J. (2021). Case Report: Neutralization of Autoantibodies Targeting G-Protein-Coupled Receptors Improves Capillary Impairment and Fatigue Symptoms After COVID-19 Infection. Front. Med..

[121] Sapkota H.R., Nune A. (2022). Long COVID from Rheumatology Perspective—A Narrative Review. Clin. Rheumatol..

[122] Gavrilova N., Soprun L., Lukashenko M., Ryabkova V., Fedotkina T.V., Churilov L.P., Shoenfeld Y. (2022). New Clinical Phenotype of the Post-Covid Syndrome: Fibromyalgia and Joint Hypermobility Condition. Pathophysiology.

[123] Phetsouphanh C., Darley D.R., Wilson D.B., Howe A., Munier C.M.L., Patel S.K., Juno J.A., Burrell L.M., Kent S.J., Dore G.J. (2022). Immunological Dysfunction Persists for 8 Months Following Initial Mild-to-Moderate SARS-CoV-2 Infection. Nat. Immunol..

[124] Glynne P., Tahmasebi N., Gant V., Gupta R. (2022). Long COVID Following Mild SARS-CoV-2 Infection: Characteristic T Cell Alterations and Response to Antihistamines. J. Investig. Med. Off. Publ. Am. Fed. Clin. Res..

[125] Schietinger A., Greenberg P.D. (2014). Tolerance and Exhaustion: Defining Mechanisms of T Cell Dysfunction. Trends Immunol..

[126] Wherry E.J., Kurachi M. (2015). Molecular and Cellular Insights into T Cell Exhaustion. Nat. Rev. Immunol..

[127] Saheb Sharif-Askari N., Saheb Sharif-Askari F., Mdkhana B., Al Heialy S., Alsafar H.S., Hamoudi R., Hamid Q., Halwani R. (2021). Enhanced Expression of Immune Checkpoint Receptors during SARS-CoV-2 Viral Infection. Mol. Ther.—Methods Clin. Dev..

[128] Zheng M., Gao Y., Wang G., Song G., Liu S., Sun D., Xu Y., Tian Z. (2020). Functional Exhaustion of Antiviral Lymphocytes in COVID-19 Patients. Cell. Mol. Immunol..

[129] Zheng H.-Y., Zhang M., Yang C.-X., Zhang N., Wang X.-C., Yang X.-P., Dong X.-Q., Zheng Y.-T. (2020). Elevated Exhaustion Levels and Reduced Functional Diversity of T Cells in Peripheral Blood May Predict Severe Progression in COVID-19 Patients. Cell. Mol. Immunol..

[130] Raman B., Cassar M.P., Tunnicliffe E.M., Filippini N., Griffanti L., Alfaro-Almagro F., Okell T., Sheerin F., Xie C., Mahmod M. (2021). Medium-Term Effects of SARS-CoV-2 Infection on Multiple Vital Organs, Exercise Capacity, Cognition, Quality of Life and Mental Health, Post-Hospital Discharge. EClinicalMedicine.

[131] Gabarre P., Dumas G., Dupont T., Darmon M., Azoulay E., Zafrani L. (2020). Acute Kidney Injury in Critically Ill Patients with COVID-19. Intensive Care Med..

[132] Lim M.A., Pranata R., Huang I., Yonas E., Soeroto A.Y., Supriyadi R. (2020). Multiorgan Failure with Emphasis on Acute Kidney Injury and Severity of COVID-19: Systematic Review and Meta-Analysis. Can. J. Kidney Health Dis..

[133] Wu H., Larsen C.P., Hernandez-Arroyo C.F., Mohamed M.M.B., Caza T., Sharshir M., Chughtai A., Xie L., Gimenez J.M., Sandow T.A. (2020). AKI and Collapsing Glomerulopathy Associated with COVID-19 and APOL 1 High-Risk Genotype. J. Am. Soc. Nephrol..

[134] Gaillard F., Ismael S., Sannier A., Tarhini H., Volpe T., Greze C., Verpont M.C., Zouhry I., Rioux C., Lescure F.-X. (2020). Tubuloreticular Inclusions in COVID-19–Related Collapsing Glomerulopathy. Kidney Int..

[135] Velez J.C.Q., Caza T., Larsen C.P. (2020). COVAN Is the New HIVAN: The Re-Emergence of Collapsing Glomerulopathy with COVID-19. Nat. Rev. Nephrol..

[136] Pan X., Xu D., Zhang H., Zhou W., Wang L., Cui X. (2020). Identification of a Potential Mechanism of Acute Kidney Injury during the COVID-19 Outbreak: A Study Based on Single-Cell Transcriptome Analysis. Intensive Care Med..

[137] Montefusco L., Ben Nasr M., D’Addio F., Loretelli C., Rossi A., Pastore I., Daniele G., Abdelsalam A., Maestroni A., Dell’Acqua M. (2021). Acute and Long-Term Disruption of Glycometabolic Control after SARS-CoV-2 Infection. Nat. Metab..

[138] Apicella M., Campopiano M.C., Mantuano M., Mazoni L., Coppelli A., Del Prato S. (2020). COVID-19 in People with Diabetes: Understanding the Reasons for Worse Outcomes. Lancet Diabetes Endocrinol..

[139] Morieri M.L., Fadini G.P., Boscari F., Fioretto P., Maran A., Busetto L., Crepaldi M.C., Vedovato M., Bonora B.M., Selmin E. (2020). Hyperglycemia, Glucocorticoid Therapy, and Outcome of COVID-19. Diabetes Res. Clin. Pract..

[140] Müller J.A., Groß R., Conzelmann C., Krüger J., Merle U., Steinhart J., Weil T., Koepke L., Bozzo C.P., Read C. (2021). SARS-CoV-2 Infects and Replicates in Cells of the Human Endocrine and Exocrine Pancreas. Nat. Metab..

[141] Brancatella A., Ricci D., Viola N., Sgrò D., Santini F., Latrofa F. (2020). Subacute Thyroiditis After SARS-CoV-2 Infection. J. Clin. Endocrinol. Metab..

[142] Vanherwegen A.-S., Gysemans C., Mathieu C. (2017). Regulation of Immune Function by Vitamin D and Its Use in Diseases of Immunity. Endocrinol. Metab. Clin. North Am..

[143] Pizzini A., Aichner M., Sahanic S., Böhm A., Egger A., Hoermann G., Kurz K., Widmann G., Bellmann-Weiler R., Weiss G. (2020). Impact of Vitamin D Deficiency on COVID-19—A Prospective Analysis from the CovILD Registry. Nutrients.

[144] Sapra L., Saini C., Garg B., Gupta R., Verma B., Mishra P.K., Srivastava R.K. (2022). Long-Term Implications of COVID-19 on Bone Health: Pathophysiology and Therapeutics. Inflamm. Res..

[145] Qiao W., Lau H.E., Xie H., Poon V.K.-M., Chan C.C.-S., Chu H., Yuan S., Yuen T.T.-T., Chik K.K.-H., Tsang J.O.-L. (2022). SARS-CoV-2 Infection Induces Inflammatory Bone Loss in Golden Syrian Hamsters. Nat. Commun..

[146] Wu Y., Guo C., Tang L., Hong Z., Zhou J., Dong X., Yin H., Xiao Q., Tang Y., Qu X. (2020). Prolonged Presence of SARS-CoV-2 Viral RNA in Faecal Samples. Lancet Gastroenterol. Hepatol..

[147] Ridruejo E., Soza A. (2020). The Liver in Times of COVID-19: What Hepatologists Should Know. Ann. Hepatol..

[148] Zhang H., Li H.-B., Lyu J.-R., Lei X.-M., Li W., Wu G., Lyu J., Dai Z.-M. (2020). Specific ACE2 Expression in Small Intestinal Enterocytes May Cause Gastrointestinal Symptoms and Injury after 2019-NCoV Infection. Int. J. Infect. Dis..

[149] Carvalho-Schneider C., Laurent E., Lemaignen A., Beaufils E., Bourbao-Tournois C., Laribi S., Flament T., Ferreira-Maldent N., Bruyère F., Stefic K. (2021). Follow-up of Adults with Noncritical COVID-19 Two Months after Symptom Onset. Clin. Microbiol. Infect. Off. Publ. Eur. Soc. Clin. Microbiol. Infect. Dis..

[150] Park S., Lee C.-W., Park D.-I., Woo H.-Y., Cheong H.S., Shin H.C., Ahn K., Kwon M.-J., Joo E.-J. (2021). Detection of SARS-CoV-2 in Fecal Samples from Patients with Asymptomatic and Mild COVID-19 in Korea. Clin. Gastroenterol. Hepatol..

[151] Yeoh Y.K., Zuo T., Lui G.C.-Y., Zhang F., Liu Q., Li A.Y., Chung A.C., Cheung C.P., Tso E.Y., Fung K.S. (2021). Gut Microbiota Composition Reflects Disease Severity and Dysfunctional Immune Responses in Patients with COVID-19. Gut.

[152] Wanner N., Andrieux G., Badia-i-Mompel P., Edler C., Pfefferle S., Lindenmeyer M.T., Schmidt-Lauber C., Czogalla J., Wong M.N., Okabayashi Y. (2022). Molecular Consequences of SARS-CoV-2 Liver Tropism. Nat. Metab..

[153] Song Q., Zhang X. (2022). The Role of Gut–Liver Axis in Gut Microbiome Dysbiosis Associated NAFLD and NAFLD-HCC. Biomedicines.

[154] Ferreira-Junior A.S., Borgonovi T.F., De Salis L.V.V., Leite A.Z., Dantas A.S., De Salis G.V.V., Cruz G.N.F., De Oliveira L.F.V., Gomes E., Penna A.L.B. (2022). Detection of Intestinal Dysbiosis in Post-COVID-19 Patients One to Eight Months after Acute Disease Resolution. Int. J. Environ. Res. Public. Health.

[155] Wang B., Zhang L., Wang Y., Dai T., Qin Z., Zhou F., Zhang L. (2022). Alterations in Microbiota of Patients with COVID-19: Potential Mechanisms and Therapeutic Interventions. Signal Transduct. Target. Ther..

[156] Yanny B., Alkhero M., Alani M., Stenberg D., Saharan A., Saab S. (2023). Post-COVID-19 Cholangiopathy: A Systematic Review. J. Clin. Exp. Hepatol..

[157] Nordvig A.S., Fong K.T., Willey J.Z., Thakur K.T., Boehme A.K., Vargas W.S., Smith C.J., Elkind M.S.V. (2021). Potential Neurologic Manifestations of COVID-19. Neurol. Clin. Pract..

[158] Guo Q., Zheng Y., Shi J., Wang J., Li G., Li C., Fromson J.A., Xu Y., Liu X., Xu H. (2020). Immediate Psychological Distress in Quarantined Patients with COVID-19 and Its Association with Peripheral Inflammation: A Mixed-Method Study. Brain. Behav. Immun..

[159] Singh S., Roy D., Sinha K., Parveen S., Sharma G., Joshi G. (2020). Impact of COVID-19 and Lockdown on Mental Health of Children and Adolescents: A Narrative Review with Recommendations. Psychiatry Res..

