# Supplementary material for: Long COVID-19 Pathophysiology: What Do We Know So Far?
Source: Microorganisms. 2023 Sep 30;11(10):2458. doi: 10.3390/microorganisms11102458 (PMC10609046; doi:10.3390/microorganisms11102458)
Supplement: Supplementary file 1 [file microorganisms-11-02458-s001.zip › microorganisms-2596295-supplementary.pdf]

Affected system and mechanisms	Ref.	Type of study	No of Patients	Comments
**Cardiovascular system**				
Endothelitis/Thrombosis	20	Position Statement	N/A	Role of vascular endothelium in cardiovascular complication
	14, 21, 31, 32	Review	N/A	Central role of endothelium in COVID-19 – Biomarkers and potential therapies
	22, 29, 30	Prospective study	210, 50, 74	Endothelial biomarkers, NETosis parameters and doppler echocardiography in COVID-19 convalescents and controls
Prolonged Inflammation	17	Prospective study	534	Study of organ function using MRI in COVID-19 convalescents
	35, 36	Prospective study	70, 318	Specific T cell responses, soluble markers of inflammation, antibody levels and neutralization capacity
Dysautonomia	37, 39	Review	N/A	Proposed model for the pathophysiology of Long Covid based on inflammatory cytokine cascades and the p38 MAP kinase signaling pathways – Hypothesis for relation of symptoms with virus-or-immune-mediated disruption of ANS
	38	Prospective study	31	Association of long covid symptoms with functionally active autoantibodies targeting G-protein coupled receptors
Dysregulation of RAAS	14,31,32, 33	Review	N/A	Review of the mechanisms by which dysregulation of the RAAS can occur and future implications for pharmacological therapies
**Respiratory system**				
Hyperinflammation/ Prolonged fibroblast activity	48, 62	Review	N/A	Literature review on the pathogenesis of lung injury - Hypothesis review on the role of neuroinflammation in long term effects of COVID-19
	61	Prospective study	7	Use of PET/CT to measure metabolic activity in inflamed organs of COVID-19 convalescents
Dysautonomia	39, 51	Review	N/A	Description of a series of long covid individuals – Hypothesis for relation of symptoms with virus-or-immune-mediated disruption of ANS and<br>proposed mechanism on persistent brainstem dysfunction
Oxidative stress	49	Review	N/A	Current evidence in post-COVID-19 pulmonary fibrosis with emphasis in histopathology, pathophysiology, tomographies
Microcirculation damage	52, 55, 56	Review	N/A	Review of long COVID-19 and hypothesized mechanisms - Physiology Society virtual meeting on understanding the current knowledge in Long-COVID
	53	Prospective study	84	Assessment of distribution of pulmonary thromboembolic events and association with severity and d-dimers
Persistent viral toxicity	57	Review	N/A	Review of proposed mechanisms in SARS-CoV-2 viral persistence and highlight of areas of research

Activation of matrix metalloproteinases	50	Review	N/A	Review on the role of matrix metalloproteinases activation in Long COVID-19
**Central Nervous System**				
BBB vulnerability	72, 74, 75	Prospective study	33, 22	Analysis of cellular-nervous micromilieu and regional mapping of consecutive olfactory nervous tracts in autopsy material – Immunostainings for ACE2 and TMPRSS2 in enteric nervous system and in choroid plexus of lateral ventricles – Quantified viral RNA in plasma samples
	73	Review	N/A	Review on the ways the virus invades the brain
Neuroinflammation/ Astrocytes and microglia activation	76, 77	Prospective study	10, 44	Profile of 65,039 single-nucleus transcriptomes from 30 frontal cortex and choroid plexus samples and autopsies to map the distribution, replication and cell-type specificity
	78, 105	Case report	N/A	Brain biopsy analysis by Western blot and qPCR for the presence of virus protein and RNA – neuropathological findings
	84	Prospective study	12	Neuropsychological and neurophysiological investigations in post-COVID-19 patients and healthy subjects
	85, 87	Review	N/A	Review of SARS-CoV-2 life cycle and approaches to suppress viral infection - role of interleukins in COVID-19 pathogenesis and their role in depression
	86	Animal Study	N/A	Determination of the effects of exogenous or endogenous IL-6 on electrically evoked postsynaptic currents on a cortical rat slice preparation
	88	Prospective study	24	Plasma from individuals recovering from COVID-19 for cytokine and antibody levels and neuronal-enriched extracellular vesicle (nEV) protein cargo analyses
	107	Meta-analysis	69	Meta-analysis for CSF and PET studies
Hypometabolism	81	Retrospective study	79	PET of long COVID patients with persistent functional complaints
	82	Prospective study	39	Longitudinal brain metabolic pattern in COVID-19-related encephalopathy using PET/CT
	83	Editorial	N/A	A hypothesis on the pathogenesis of long-term neuropsychiatric COVID-19
Immune dysregulation	90	Review	N/A	Discusssion of eleven cases of GBS regarding symptoms and cross-reactivity of COVID spike proteins with nerve glycolipids
	91	Letter to the Editor/Prospective study	216	Coagulation tests and lupus anticoagulant in COVID-19 patients and comparison with healthy controls
	92	Cross-sectional study	19	Patients admitted to ICU were studied for routine hemostasis tests, natural anticoagulants, coagulant factors and antiphospholipid antibodies
Hypercoagulopathy	93, 95	Correspondence/Review	13	Postmortem MRI of the brains of patients with COVID-19 and histopathological examination focused on microvascular changes in the

				olfactory bulb and brain stem and review no MRI brain findings of 126 patients
	94	Retrospective study	23	Patients with acute CVD including histologic samples
	96	Prospective study	81	Assessment of endothelial cell markers, platelet activation markers, coagulation factors, endogenous anticoagulants and fibrinolytic enzymes
**Immune System**				
Immune dysregulation	110, 112, 113, 114, 116	Review	N/A	Production of antibodies against the virus and ACE-2, interferon, causing GB and thrombocytopenia
	111, 115, 117, 119	Prospective study	100/106/215/13	Original research on measurement of ANA and immunoglobulin signature, based on IgM and IgG3 levels and ACE2 antibodies in COVID-19 patients
	122, 123	Review/Case Report	N/A	Patients developing MAS and development of autoimmune diseases in long COVID
	124, 125, 129, 130	Letter/Prospective studies	62/65/68/16	Studies showing prolonged activation of T-cells and expression of exhaustion markers
**Other Systems**				
Kidneys	132, 136, 134, 135	Review/ Case series	N/A/6	Review on epidemiology and potential mechanisms related to AKI<br>Investigation of genetic, histopathologic and molecular features in COVID-19 patients presented with AKI, nephrotic-range proteinuria and collapsing glomerulopathy
	137	Prospective study	15	Study based on single-cell transcriptome analysis proposing a potential mechanism of AKI
Metabolic	139, 143, 145	Review	N/A	Review on potential reasons for worse outcomes in diabetes patients and mechanisms of vitamin D regulation of the immune system and implications on bone health
	141	Prospective study	5	Postmortem full-body examinations showing human pancreas as a target of SARS-CoV-2 infection
	144	Observational study	109	Investigation of associations of vitamin D status to COVID-19 presentation
	146	Animal study	N/A	Effects of COVID-19 on bone metabolism in hamster model
GIS	149	Metanalysis	466	ACE2 expression in different human organs and relationship with clinical patterns
	147, 151	Prospective study	98/46	Gastrointestinal symptoms and shedding of virus into feces
	153	Prospective study	99	Clinical, histopathological, molecular and bioinformatic evidence for hepatic tropism of SARS-CoV-2
	154, 156	Review	N/A	Summary of the evidence of gut microbiota dysbiosis and microbial metabolites changes in NAFLD and NAFLD-HCC and COVID-19
	152, 155	Prospective study	100/149	Evaluation of intestinal microbiota in different post-COVID-19 periods and correlation with clinical data

Table S1. Summary of the most established mechanisms for the symptoms of long-COVID-19 as supported by published evidence.
